# Proteomic and Transcriptional Profiles of Human Stem Cell-Derived β Cells Following Enteroviral Challenge

**DOI:** 10.3390/microorganisms8020295

**Published:** 2020-02-20

**Authors:** Julius O. Nyalwidhe, Agata Jurczyk, Basanthi Satish, Sambra Redick, Natasha Qaisar, Melanie I. Trombly, Pranitha Vangala, Riccardo Racicot, Rita Bortell, David M. Harlan, Dale L. Greiner, Michael A. Brehm, Jerry L. Nadler, Jennifer P. Wang

**Affiliations:** 1 Department of Microbiology and Molecular Cell Biology and Leroy T. Canoles Jr. Cancer Research Center, Eastern Virginia Medical School, Norfolk, VA 23501, USA; NyalwiJO@EVMS.EDU (J.O.N.); jnadler@nymc.edu (J.L.N.); 2 Program in Molecular Medicine, University of Massachusetts Medical School, Worcester, MA 01655, USA; agata.jurczyk@umassmed.edu (A.J.); sambra.redick@umassmed.edu (S.R.); bortell1@comcast.net (R.B.); dale.greiner@umassmed.edu (D.L.G.); michael.brehm@umassmed.edu (M.A.B.); 3 Department of Medicine, University of Massachusetts Medical School, Worcester, MA 01655, USA; basanthi.satish@umassmed.edu (B.S.); natasha.qaisar@umassmed.edu (N.Q.); melanie.trombly@umassmed.edu (M.I.T.); riccardo.racicot@gmail.com (R.R.); David.Harlan@umassmemorial.org (D.M.H.); 4 Department of Bioinformatics and Integrative Biology, University of Massachusetts Medical School, Worcester, MA 01655, USA; pranitha.vangala@umassmed.edu; 5 Department of Medicine and Pharmacology, New York Medical College, Valhalla, NY 10595, USA

**Keywords:** coxsackie B virus, beta cells, type 1 diabetes

## Abstract

Enteroviral infections are implicated in islet autoimmunity and type 1 diabetes (T1D) pathogenesis. Significant β-cell stress and damage occur with viral infection, leading to cells that are dysfunctional and vulnerable to destruction. Human stem cell-derived β (SC-β) cells are insulin-producing cell clusters that closely resemble native β cells. To better understand the events precipitated by enteroviral infection of β cells, we investigated transcriptional and proteomic changes in SC-β cells challenged with coxsackie B virus (CVB). We confirmed infection by demonstrating that viral protein colocalized with insulin-positive SC-β cells by immunostaining. Transcriptome analysis showed a decrease in insulin gene expression following infection, and combined transcriptional and proteomic analysis revealed activation of innate immune pathways, including type I interferon (IFN), IFN-stimulated genes, nuclear factor-kappa B (NF-κB) and downstream inflammatory cytokines, and major histocompatibility complex (MHC) class I. Finally, insulin release by CVB4-infected SC-β cells was impaired. These transcriptional, proteomic, and functional findings are in agreement with responses in primary human islets infected with CVB ex vivo. Human SC-β cells may serve as a surrogate for primary human islets in virus-induced diabetes models. Because human SC-β cells are more genetically tractable and accessible than primary islets, they may provide a preferred platform for investigating T1D pathogenesis and developing new treatments.

## 1. Introduction

Type 1 diabetes (T1D) is an inflammatory autoimmune disease characterized by the destruction of pancreatic β cells. Although the etiology of T1D is not fully understood, enteroviral infections have long been suspected to be a contributing factor [1,2,3,4]. Viral infections can explain the increasing prevalence of diabetes and seasonal variation in onset. Coxsackie viral antigens can be detected in pancreatic tissues from subjects with T1D [3], and serum viral RNA is more frequently reported in T1D cases even before the detection of autoantibody [5,6], indicating that viral infection may participate early in T1D pathogenesis. Human pancreatic islet cells express the coxsackie and adenovirus receptor (CAR), and significant β-cell stress and damage may occur following viral infection, as evidenced in samples from T1D and autoantibody-positive subjects [2,7]. In addition, human islet autoimmunity is associated with prolonged coxsackievirus infection [8]. Genome-wide association studies show associations between the risk for human T1D and polymorphisms in interferon (IFN) response genes in the general population [4,9,10,11]. We and others have reported that inflammatory cytokines and type I IFN pathways are induced following treatment of human islets with coxsackie B virus (CVB)4 ex vivo [12,13,14] and engrafted human islets in vivo [15]. Altogether, such findings suggest a potential relationship between virus infection, IFN induction, and development of autoimmunity.

Human pluripotent stem cells (hPSC), including the human embryonic stem cell (hESC) line HUES8, can be differentiated to an endocrine phenotype through treatments with cocktails of small molecule agonists and growth factors [16]. These stem cell-derived β (SC-β) cells are comprised of insulin-producing β cells that can respond to high-glucose challenges and depolarization with potassium chloride, and additional hormone-positive cells are represented in the cell clusters, including glucagon-producing α cells. Because they are differentiated from clonal progenitor cells, SC-β cells may provide an accessible platform for diabetes therapeutic development and disease modeling. 

Our goal was to determine whether human SC-β cells can serve as a model to examine how viral infection may contribute to T1D. After confirming that human SC-β cells can be infected with CVB, we defined the proteomic, transcriptional, and functional responses following viral infection. Here, we report that CVB infection of SC-β cells induces strong innate immune responses, transcriptional decreases in insulin, and impaired insulin release. 

## 2. Materials and Methods 

### 2.1. Cells

Human SC-β cells were derived from the embryonic stem cell line HUES8, as previously described [16], and were generously provided by Dr. Douglas Melton, Harvard University. Primary human islets from an adult donor without diabetes were obtained from Prodo Laboratories, Inc. (Aliso Viejo, CA 92656, USA). The donor was #HP-16120-01, a 33-year-old Caucasian male with a BMI of 22.7 kg/m^2^ with no history of diabetes, and Hgb A1c of 5.1%. The patient died from head trauma caused by a motor vehicle accident. Islets were 95% pure.

### 2.2. Infection

SC-β cells were plated at 5 × 10^5^ cells/well in a 12-well plate. Cells were either uninfected or infected with CVB4-JVB (ATCC, Manassas, VA 20110, USA; VR-194). Virus was propagated in HeLa cells (see [15]) and added at a multiplicity of infection (MOI) of 10 (based on cell counts of dissociated clusters) in 200 μL of media CMRL 1066 containing 1 g/L (5 mM) D-glucose (Gibco 11530-037, Thermo Fisher Scientific, Pittsburgh, PA 15205, USA) + 10% fetal bovine serum (FBS) + L-glutamine + penicillin/streptomycin, and incubated at 37 °C for 1 h. Next, the cells were supplemented with additional media for a final volume of 500 μL/well without removing the initial virus inoculum. Embedding was performed as described in [17]; in brief, cells were washed twice with phosphate-buffered saline (PBS) then spun at 1000 rpm, then fixed in 500 µL of 10% formalin for 30 min. Cells were spun down and resuspended in 500 µL PBS with agarose blue beads. Cells were again spun down at 1000 rpm for 3 min, then resuspended in warm Histogel (75 µL), and the gel containing islets was transferred to a glass slide on ice; the resulting gel button was paraffin embedded. Cells were also collected at 24, 48, and 72 h for total RNA and protein. Primary human islets were plated and infected as previously described [12], with cells being collected at 72 h for protein.

### 2.3. Immunofluorescence

Antigen retrieval was mediated at 98 °C for 45 min in formalin-fixed, paraffin-embedded sections. Sections were blocked with PBS containing 1% BSA and 5% normal goat serum, then incubated with the following primary antibodies overnight: guinea pig antibody to insulin (1:150; Dako, Agilent, Santa Clara, CA 95051, USA); rabbit antibody to glucagon (1:50; Dako); and mouse antibody to viral protein (VP1), clone 5-D8/1 (1:500; Dako). Sections were incubated for 1 h with the following secondary antibodies at 1:1000 dilution: Alexa Fluor-488 goat antibody to guinea pig IgG; Alexa Fluor-647 donkey antibody to rabbit IgG; and Alexa Fluor-594 goat antibody to mouse IgG (catalog #A11073, #A31573, and #A11032, respectively; Life Technologies, Thermo Fisher Scientific). DNA was stained with Hoechst 33,342 at 1 µg/mL (Millipore Sigma, St. Louis, MO 63178, USA). Sections were mounted with ProLong Diamond Antifade Mountant (Life Technologies, Thermo Fisher Scientific). Images were acquired with a Nikon Eclipse Ti series microscope and analyzed with NIS-Elements Imaging software, version 4.13.04. The colocalization tool in NIS-Elements was used to measure the Mander’s overlap coefficient (k1) following automated background subtraction for all images.

### 2.4. Perifusion

A total of 25 clusters were hand-selected from three wells each of CVB-infected and uninfected cells at 48 h post-infection and loaded for perifusion using a Biorep PERI 4.2 perifusion system (Biorep Technologies, Miami, FL, 15804, USA). The islets were perifused in Kreb’s buffer (115 mM NaCl, 5 mM KCl, 24 mM NaHCO_3_, 1 mM MgCl_2_, 2.2 mM CaCl_2_ at pH 7.4) supplemented with 0.17% bovine serum albumin and 2.5 mM glucose (basal), followed by 20 mM glucose, 20 mM glucose + 10 nM exendin-4 (Tocris Bioscience catalog #1933, Bio-Techne, Minneapolis, MN 55413, USA), then 20 mM KCl with 2.5 mM glucose. Medium was collected at a flow rate of 100 μL/min to assess insulin secretion. Insulin concentration was measured using an insulin ELISA kit (Alpco catalog #80-INSHUE10.1, Salem, NH 03079, USA). The islets were collected at the end of the perifusion and placed in acidified ethanol overnight to determine total insulin levels. 

### 2.5. Viability Studies

Three wells of CVB-infected and uninfected cells were collected at 48 and 72 h post-infection, washed with Hanks’ buffered salt solution, spun at 900 rpm for 3 min, washed with 5 mM EDTA, and spun again, and then 1 mL warm Accutase cell detachment solution (Innovative Cell Technologies, San Diego, CA 92121, USA) was added. Cells were pipetted up and down 10 times and then incubated at 37 °C for 3 min. This was repeated until single cells were observed by microscopy. A total of 2% BSA in PBS was added to cells to stop the enzymatic reaction, and cells were spun at 1000 rpm for 4 min, then washed once with PBS. Cells were resuspended in PBS and stained with FITC-annexin V and 7-amino-actinomycin D (7-AAD) according to the manufacturer’s directions (BD Biosciences, San Jose, CA 95131, USA). At least 20,000 cells per sample were analyzed using a FACSCalibur (BD Biosciences) and FlowJo software Version 10 (BD Biosciences).

### 2.6. Proteomics

Samples were processed as previously described [12]. In brief, SC-β cells were harvested and washed three times with PBS prior to processing for mass spectrometry. Pelleted cells were completely solubilized in 50% trifluoroethanol in 50 mM triethyl ammonium bicarbonate. Protein concentrations were determined by BCA assay and processed for LC–MS/MS, as we have previously described [18]. Each sample was analyzed in triplicate. Protein identification and comparative label-free quantitative analysis was performed using MaxQuant and Andromeda [19,20]. Protein Group.txt files from MaxQuant were imported to Perseus for statistical analysis using the label-free quantitation (LFQ) intensities from MaxQuant [21,22]. Differentially expressed proteins were used for ingenuity pathway analysis (IPA; see Ingenuity Systems, http://www.ingenuity.com; and Qiagen, https://www.qiagenbioinformatics.com/products/ingenuity-pathway-analysis/) (Germantown, MD 20874, USA) to characterize the molecular functions of differentially expressed proteins between the mock- and CVB4-infected SC-β cells. 

### 2.7. Transcriptome (RNA-Seq)

Total RNA was extracted from TRIzol. RNA-Seq libraries were prepared from 10 ng of starting RNA using SMART-Seq. Pooled libraries were sequenced for 2 × 75 cycles to obtain paired end reads using a NextSeq 500 (Illumina, San Diego, CA 92122, USA). *Trimmomatic-0.32* was used to remove 5′ or 3′ stretches of bases having an average quality of less than 20 in a window size of 10. Only reads longer than 36 bases were kept for further analysis. *RSEM* v1.2.28 was used to estimate gene expression, with parameters *-p 4-bowtie-e 70-bowtie-chunkmbs 100-strand-specific*. Gene quantification was run on the transcriptome (RefSeq v69 downloaded from UCSC Table Browser). The CVB4 reference sequence is GenBank X05690.1. Genes with more than 10 transcripts per million (TPM) in any time point were considered expressed, and genes that did not achieve this threshold were removed from further analysis. The expressed genes were then ranked by their coefficient of variation (decreasing) and the top 1000 genes were determined. For the heatmap, *k*-means clustering was performed on the data.

The gene ontology (GO) enrichments tests were performed on this subset of 1000 genes with all the expressed genes set as background. Sequences are available at GSE145074.

### 2.8. Statistics

All statistics were calculated using Prism Version 8.0 (GraphPad, San Diego, CA 92108, USA). 

## 3. Results

### 3.1. Immunostaining Revealed Colocalization of Coxsackie Viral Protein and Insulin in SC-β Cells

**Figure 1:**
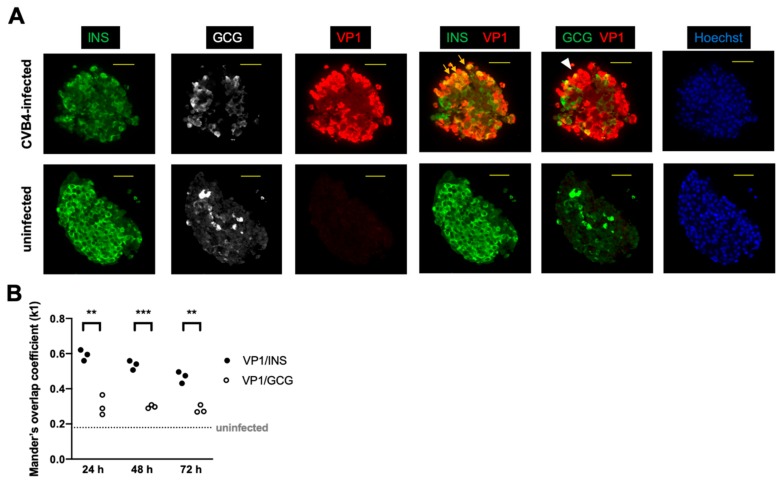
Immunostaining of stem cell-derived β (SC-β) cells revealed abundant insulin (INS)- and glucagon (GCG)-positive cells with viral protein (VP1) predominantly colocalizing with INS. (**A**) The colocalization of green and red is shown as yellow. Arrows show examples of colocalization between VP1 and INS, and the arrowhead shows colocalization between VP1 and GCG. A representative cell cluster from each condition at 48 h post-infection with coxsackie B virus (CVB)4 (or control uninfected) is shown. INS = green, GCG = white or green, VP1 = red, Hoechst (DNA stain) = blue. Scale bar = 50 μm. (**B**) Mander’s overlap coefficients (*k1*) for VP1 and INS and for VP1 and GCG in infected cells are shown at the indicated time points following infection. VP1 and INS colocalized more frequently than VP1 and GCG. (** *p* < 0.01; *** *p* < 0.001, multiple *t*-test). The coefficient was significantly lower for uninfected controls for which the median value is shown as the horizontal dashed line (*p* < 0.0001, Mann–Whitney test). Each point represents one field of view.

We first determined that coxsackie virus can infect human SC-β cells. Human SC-β cells derived from the embryonic stem cell line HUES8 were challenged with CVB4 strain JVB in vitro, and cells were harvested up to 72 h post-infection. Following infection, cells were embedded for immunostaining. Samples collected at 48 and 72 h were examined in two independent experiments with representative immunostaining shown in Figure 1. Viral protein colocalized with insulin protein, indicating successful infection of insulin-positive cells. CVB4 also colocalized with some of the glucagon-expressing cells, albeit with lower frequency than with insulin-positive cells. 

### 3.2. Proteomic Analysis Revealed Activation of Inflammatory Pathways, IFN, and MHC Class I in SC-β Cells over Time, with Comparative Analysis with Proteomics of Primary Human Islets

To assess the proteomic profile of CVB4-infected SC-β cells, we performed liquid chromatography–tandem mass spectrometry (MS) proteomic assessments at three time points following infection, comparing CVB4-infected cells to control uninfected cells. Using software algorithms to identify and quantify differential protein expression, we identified a total of 2894 proteins in our samples. Of these differentially expressed proteins, 1895 proteins were quantifiable on the basis of a robust inclusion filtering criteria. At 24 h, the coxsackie viral protein (POLG_CXB4J) was the only differentially increased protein (Table 1). At 48 h post-infection, viral protein remained increased, and additional differentially expressed proteins were present, including MX1 and CXCL6 (Table 1). At 72 h, interferon-stimulated genes (ISG) including *MX1*, *IFIT1*, *IFIT3*, *DDX58*, and *STAT1* and chemokines such as CXCL1 and CXCL8 were induced. Wolframin (WFS1) was decreased at 72 h; its loss is reportedly associated with endoplasmic reticulum (ER) stress and β-cell dysfunction [23] (see Table S1). Of note, class I MHC molecules including HLA-A and HLA-B, as well as the antigen-processing proteins B2M and TAP1 [24], were all increased following CVB infection. We previously reported such proteomic changes in cultured primary human islets infected with CVB [12]. 

**Figure 2:**
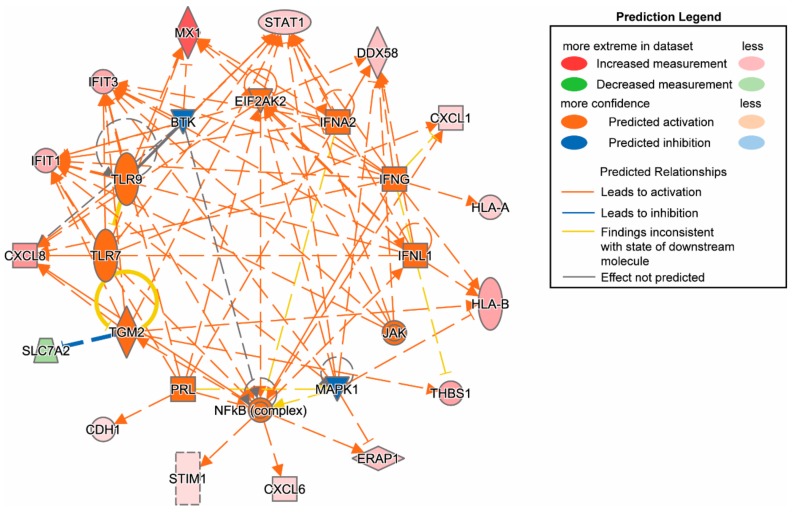
Ingenuity pathway analysis of SC-β cells infected with CVB4 for 48 h (proteomics). Inflammatory and type I IFN pathways were activated.

We used upstream regulator analysis and ingenuity pathway analysis (IPA) to analyze SC-β cells with and without CVB infection. At 48 h, cytokines and transcriptional regulators were highly induced by infection (Figure 2), and several upstream regulators were significantly activated at 72 h (Table S1). In our previously published mass spectrometry analysis of cultured primary human islets following CVB4 challenge, we identified proteins that were significantly differentially regulated following CVB4 infection at 48 h [12]. Here, we compared IPA data from SC-β cells with that from primary human islets at baseline (uninfected) and following CVB4 infection for 72 h (Table 2) to determine differential activated pathways (upstream activation) through protein expression profiles. We observed overlap in activated/inhibited pathways and the molecules involved for CVB4-infected SC-β cells and primary human islets. These included the shared upstream regulators TLR7, PRL, IFNL1, TGM2, TLR9, IFNG, IFNA2, and EIF2AK2. Thus, key proteomic changes observed in CVB4-infected human islets were found to be replicated in CVB4-infected SC-β cells. Levels of CVB polyprotein (POLG_CXB4J) were similar between SC-β cell and human islet experiments at 48 or 72 h post infection, as we previously found that infecting 100 primary human islet equivalents (~ 1 × 10^5^ cells) with 10^6^ plaque-forming units (PFU) of CVB4 yielded a 8.1-fold increase in the viral protein CXB4 at 48 h [12]; likewise, we observed a 9.0-fold increase in CXB4 at 48 h with infection of 5 × 10^5^ SC-β cells with 5 × 10^6^ PFU of CVB4. Although the MOI of 10 used in both primary human islet and SC-β cell experiments was quite high, proteomic changes and levels of infection were consistent between these two models.

### 3.3. Transcriptome Analyses of CVB4-Infected SC-β Cells Showed a Temporal Increase in Viral Transcripts and Decreases in Genes Associated with the β Cell Phenotype

**Figure 3:**
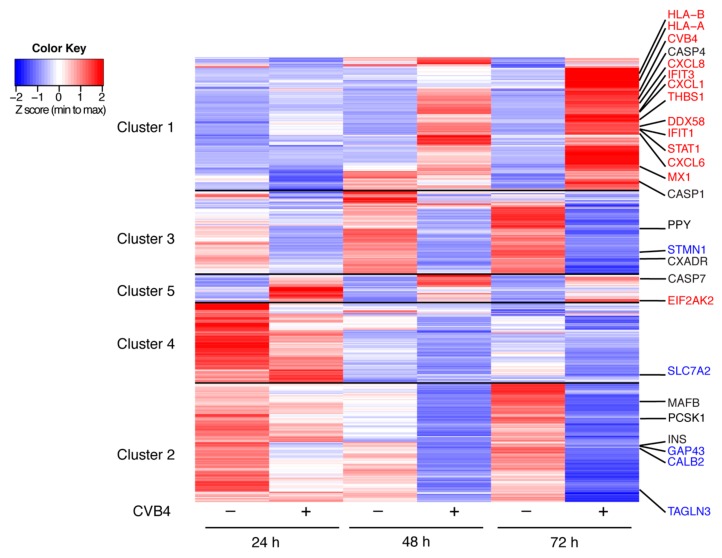
RNA-Seq heatmap showing the top 1000 genes differentially expressed in SC-β cells following CVB4 infection. The genes were filtered to have at least 10 transcripts per million (TPM) at any time point. The 1000 most changing genes were then clustered using *k*-means; within each cluster, genes were ordered using hierarchal clustering. Specific gene names are shown in red if increased during infection by both proteomic and transcriptome analyses, in blue if decreased with infection by both analyses, and in black if notable transcriptome changes occurred in the absence of significant proteomic changes. Five clusters are depicted and full gene lists are available in Table S2.

RNA-Seq of CVB4-infected SC-β cells was performed on samples collected at 24, 48, and 72 h post-infection. The top thousand modulated transcripts are presented in the heatmap in Figure 3. We highlight the top thousand differentially expressed genes/transcripts that were also significantly modulated by proteomic analysis in the heatmap (Figure 3). All transcripts quantified as transcripts per million (TPM) are available in Table S2. *CVB4* transcripts nearly doubled between 24 and 72 h, suggesting active replication in these cells at this infectious dose. Transcriptome analysis of SC-β cells revealed decreases in expression of several β cell-associated genes during CVB4 infection. *INS*, which is highly expressed at baseline in SC-β cells, decreased by >5-fold following CVB4 infection (Figure 3). Transcripts for canonical β-cell markers including *PDX1, FOXA2*, *G6PC2*, and *PAX6* were present at low levels and also decreased following infection but were not amongst the top 1000 differentially expressed transcripts (see Table S2). Other transcripts that we previously reported as being β-cell specific [25], including *PCSK1* and *MAFB* (Figure 3), and *GLIS3* and *CASR* (Table S2)*,* all decreased with infection. In contrast, α-cell-specific transcripts [25] including *GCG*, *ARX*, *BAMBI*, *DPP4*, and *POU6F2* were not affected by infection (see Table S2), which corresponded with our observation that viral infection was infrequent in α cells (see Figure 1). The δ-cell marker gene *SST* was initially lower in infected compared to uninfected cells, but no difference was seen at 72 h. The γ-cell gene *PPY* decreased with infection over the time course. Finally, infection resulted in a nearly 10-fold decrease in expression of *CXADR*, which encodes CAR, the receptor for the coxsackie virus. This finding is congruent with reported data for explanted human islets following CVB infection [7].

**Figure 4:**
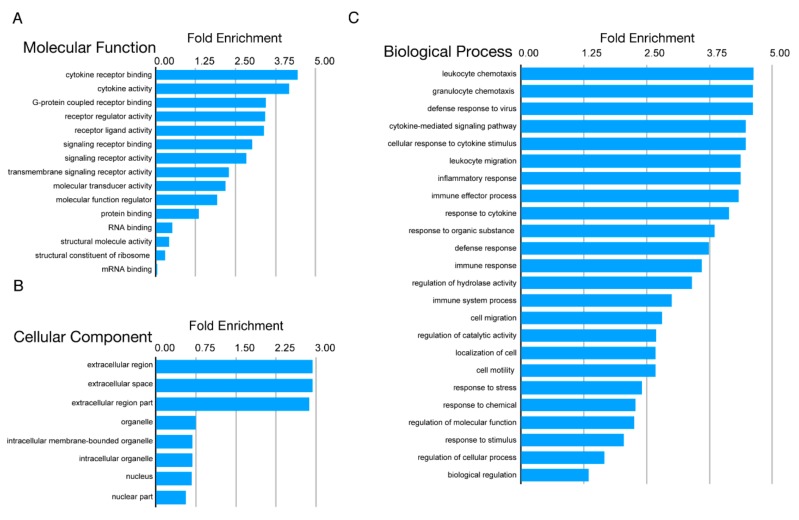
Gene ontology (GO) analysis of most variable genes 24, 48, and 72 h post CVB infection. Figure shows the GO terms for the top 1000 differentially expressed genes identified by RNA-Seq. GO terms are divided into three categories (**A**) molecular function, (**B**) cellular component, and (**C**) biological process.

Examination of other modulated transcripts (Figure 3) revealed the activation of innate immune pathways, including those involved in type I IFN signaling, cytokine-mediated signaling, and MHC class I. Gene ontology (GO) enrichment revealed that the majority of the pathways related to innate immune signaling were enriched with CVB infection (Figure 4); Figure 4C shows strong enhancement of the biological processes for chemotaxis, defense response to virus, cytokine responses, and inflammation. This corresponded with the proteomics IPA findings for infected SC-β cells.

### 3.4. Perifusion Studies Showed Impaired Insulin Release by CVB4-Infected Cells

**Figure 5:**
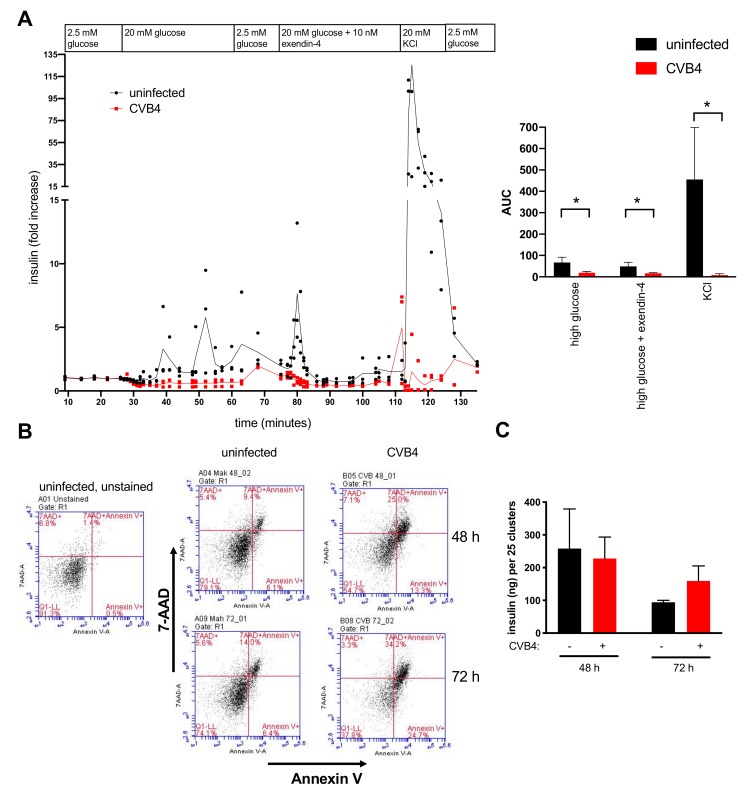
Assessment of perifusion, cytotoxicity, and total insulin in CVB4-infected SC-β cells. (**A**) Cells were examined by perifusion analysis at 48 h post-infection to measure insulin secretion. The data were normalized to basal insulin secretion values measured prior to challenge with high glucose. Insulin secretion in response to high glucose (20 mM), high glucose plus exendin-4 (10 nM), and KCl (20 mM) was diminished in CVB4-infected cells compared to uninfected cells (left panel). Perifusion data are also presented as the area under the curve (AUC) for insulin secretion under conditions of high glucose (27–60 min), high glucose + exendin-4 (77–108 min), and KCl (112–121 min) (right panel). The mean ± SD for triplicate samples is shown. * *p* < 0.05, Student’s *t*-test. (**B**) Flow cytometry of cells stained with annexin V and 7-aminoactinomycin D (7-AAD) showed 2.7-fold and 2.4-fold increases in late apoptotic populations (i.e., annexin V+ and 7-AAD+) in infected compared to uninfected cells at 48 h and 72 h post-infection, respectively. (**C**) Total insulin content did not differ between uninfected and CVB4-infected cells at either 48 or 72 h. Error bars indicate the SD of values from triplicate wells measured in duplicate. Differences between uninfected and infected cells were not statistically significant. One of two replicate experiments is shown for each panel, with the replicate experiment having similar trends (see Figure S1).

To assess responsiveness of cells following infection, we conducted perifusion studies. A significant decrease in glucose-stimulated insulin secretion and glucose + exendin-4-stimulated insulin secretion was apparent in CVB4-infected SC-β cells compared to uninfected cells (Figure 5A). Perifusion also showed a large release of insulin by uninfected cells upon challenge with the secretagogue potassium chloride (KCl), whereas the readily releasable pool of granules was lost in CVB4-infected cells. These coxsackievirus-induced alterations in insulin release were statistically significant (Figure 5A, right panel) and consistent with findings reported in cultured human islets [26]. 

To determine if the decrease in insulin secretion was in part a consequence of virus-induced SC-β cell death, we measured cell viability at 48 and 72 h post-infection using annexin V and 7-amino-actinomycin D (7-AAD) staining and flow cytometry to measure apoptosis and necrosis, respectively. Total cell death (i.e., annexin V^+^/7-AAD^+^) was increased ~2.5 fold in CVB4-infected cells compared to uninfected cells, specifically 25.0% versus 9.4% (48 h) and 34.2% versus 14.0% (72 h) (Figure 5B). This was in agreement with our finding that the pro-apoptotic genes *CASP1*, *CASP4*, and *CASP7* were increased during infection (see Figure 3). Total insulin content did not significantly differ between CVB4-infected and uninfected SC-β cells at either time point (Figure 5C), which corresponded to our proteomics findings. In summary, although CVB4 infection was associated with some SC-β cell death, which may account for some of the decreased *INS* transcripts and insulin responsiveness, the total insulin levels were not significantly altered. 

## 4. Discussion

Human stem cell-derived SC-β cells develop into islet-like clusters comprised of cells that contain mainly insulin and, to a lesser extent, glucagon and somatostatin [14], and thus provide a unique opportunity to study diabetes in a human-derived cell culture system resembling native human islets. SC-β cells have been shown to have α- and β-cell markers in a defined distribution, with 32–34% of cells being C-peptide+, 8–9% GCG+, 5–6% SST+, 8% C-peptide+/GCG+, 5% C-peptide/+SST, and 2% GCG+/SST+ [16]. We showed that SC-β cells, including insulin-positive cells, can be infected with CVB4 to study the etiology of virus-induced diabetes. Infection was confirmed with three independent methods, namely, by immunostaining, by quantifying increases in viral transcripts over time, and through proteomic measurement of viral polyprotein. The virus inoculum used in these studies is comparable to titers reported in human infection [27] and in the mouse pancreas [28]. Transcriptome and proteome profiles during CVB4 infection over time revealed robust activation of innate immune pathways, including inflammatory cytokines and chemokines, and are consistent with our reported observations in CVB4-infected primary human islets [12] and with human T1D data [29,30], including our recent transcriptome analysis of islet β cells from T1D donors [31]. 

We observed a decline in *INS* and other β-cell phenotype transcripts and decreases in insulin secretion following CVB4 infection of SC-β cells. By 72 h post-infection, cytotoxicity of insulin-positive cells doubled and *INS* transcripts decreased by >5 fold, yet total insulin levels were comparable between CVB4-infected and uninfected cells. Together, these data suggest that toxicity, transcriptional changes, and insulin secretion all contributed to β-cell dysfunction during infection. Future experiments are needed to determine if the overall decrease in *CXADR* (the gene that encodes the receptor for coxsackievirus) during infection is transcriptionally regulated or is a consequence of virus-mediated cell death. Death of insulin-producing β cells, although present, may not account for all the transcriptional changes that follow infection, consistent with the concept that β cells remain viable but lose function early in the course of T1D onset [32]. In the future, studies at the single-cell level may better define β-cell-specific events during viral infection.

In a previous study, we found that mRNA for both MHC class I and II were increased in β cells from T1D donors compared to non-diabetic donors [31]. A recent study revealed that inflammation and ER stress impacted peptide presentation by MHC class I in human β cells [33]. Mechanisms of MHC class I peptide presentation could be further explored using SC-β cells. In the current study and in our prior report on primary human islets [12], MHC class II molecules were not increased following CVB4 infection. Of note, immunostaining of pancreatic samples in the T1D study showed increases in MHC class II proteins only in islets with lymphocytic infiltration [31], and thus the presence of additional cell types may be required for MHC class II upregulation. 

Our studies indicate that human SC-β cells can potentially act as a surrogate for primary human islets for understanding virus-induced mechanisms of β-cell dysfunction. SC-β cells engrafted in immunodeficient mice could be studied in virus models in vivo to interrogate T1D pathogenesis as well as to help identify new treatments to maintain β-cell functional mass. In future studies, SC-β cells derived from either T1D patients [34] or genetically susceptible individuals may be used to interrogate how these cells might respond differently to environmental perturbants (such as virus infection), given that environmental factors are thought to contribute to T1D development. SC-β cell experiments in vitro or in vivo (i.e., transplanted into mice) could be used to address specific hypotheses on the role of β cells in T1D development. For example, do individuals who are genetically predisposed to develop T1D have an enhanced inflammatory response to virus infection? Are β cells from T1D patients more prone to apoptosis? Finally, as they are more genetically tractable than primary islets, SC-β cells could serve as a platform for investigating T1D pathogenesis with adeno-associated virus knockdown or with CRISPR-induced knockout of target genes. 

## Figures and Tables

**Table 1:** Proteins with >1.5-fold (log_2_) difference in CVB4-infected compared to uninfected SC-β cells at 24 and 48 h.

**24 h**			
**= -LOG** **(*P*-value)**	**Fold Difference (log2 transformed)**	**Protein IDs**	**Gene name/Protein name**
7.897847	9.588954	P08292	*POLG_CXB4J*/Genome polyprotein
3.013728	−2.054616	Q99816	*TSG101*/Tumor susceptibility gene 101 protein
3.153537	−2.260496	P30519	*HMOX2*/Heme oxygenase 2
2.616789	−2.945653	P43307	*SSR1*/Translocon-associated protein subunit alpha
**48 h**			
**= -LOG** **(*P*-value)**	**Fold Difference (log2 transformed)**	**Protein IDs**	**Gene name/Protein name**
3.789608	9.046398	P08292	*POLG_CXB4J*/Genome Polyprotein
3.216631	4.741543	P20591	*MX1*/Interferon-induced GTP-binding protein Mx1
3.350592	4.65571	P12955	*PEPD*/Xaa-Pro dipeptidase
3.742026	3.205037	Q04941	*PLP2*/Proteolipid protein 2
5.440948	3.004789	P62072	*TIMM10*/Mitochondrial import inner membrane translocase subunit Tim10
6.367597	2.369012	P07996	*THBS1*/Thrombospondin-1
4.888743	2.081825	P80162	*CXCL6*/C-X-C motif chemokine 6
1.863447	1.912146	Q6KCM7	*SLC25A25*/Calcium-binding mitochondrial carrier protein SCaMC-2
3.944867	1.822556	P10145	*CXCL8*/Interleukin-8
2.789387	1.807034	O95786	*DDX58*/Probable ATP-dependent RNA helicase DDX58
3.484639	1.529275	P09914	*IFIT1*/Interferon-induced protein with tetratricopeptide repeats 1
2.11946	1.525606	P30480	*HLA-B*/HLA class I histocompatibility antigen
5.962474	−1.66812	P22676	*CALB2*/Calretinin
4.316548	−1.70801	P67809	*YBX1*/Nuclease-sensitive element-binding protein 1
3.94315	−1.72381	Q13310	*PABPC4*/Polyadenylate-binding protein 4
2.155246	−1.80032	P30519	*HMOX2*/Heme oxygenase 2
2.691352	−1.98576	Q9UMX0	*UBQLN1*/Ubiquilin-1
1.847708	−2.11509	Q9UHD9	*UBQLN2*/Ubiquilin-2
3.702343	−2.17211	Q9Y2W2	*WBP11*/WW domain-binding protein 11
3.326408	−2.22052	Q9NX14	*NDUFB11*/NADH dehydrogenase [ubiquinone] 1 beta subcomplex subunit 11
3.449024	−2.28579	P50402	*EMD*/Emerin
2.690032	−2.39827	Q9UNH7	*SNX6*/Sorting nexin-6;Sorting nexin-6, N-terminally processed
3.795319	−2.47833	Q9H3P7	*ACBD3*/Golgi resident protein GCP60
2.292801	−3.22337	P43307	*SSR1*/Translocon-associated protein subunit alpha

**Table 2:** Proteomics. Differentially regulated genes in CVB4-infected SC-β cells compared to primary beta cells (results shown are for 72 h post infection).

Upstream Regulator	Molecule Type	Predicted Activation State	Activation z-Score	*p*-Value of Overlap	Target Molecules in Dataset
IFN-α	group	Activated	2.369	3.04E-11	APOL2, EIF2AK2, GBP1, IFIT1, IFIT2, IFIT3, ISG15, PML
TLR7	transmembrane receptor	Activated	2.433	5.28E-09	ICAM1, IFIT1, IFIT3, ISG15, MX1, STAT1
PRL	cytokine	Activated	2.236	1.88E-07	EIF2AK2, IFIT1, IFIT3, ISG15, SAMHD1
JAK1	kinase	Activated	2	2.32E-08	HLA-A, IFIT2, MX1, STAT1
IL27	cytokine	Activated	2.414	5.44E-10	B2M, HLA-A, HLA-B, ICAM1, STAT1, TAP1
IFNL1	cytokine	Activated	3.113	4.56E-18	EIF2AK2, GBP1, IFIT1, IFIT2, IFIT3, ISG15, MX1, PML, STAT1, UBE2L6
TNF	cytokine	Activated	2.706	5.82E-08	GBP1, HLA-B, ICAM1, IFIT3, PML, STAT1, TAP1, TAPBP, TYMP
TGM2	enzyme	Activated	2.646	4.99E-08	HLA-B, IFIT1, IFIT2, IFIT3, SAMHD1, STAT1, TAP1
TLR9	transmembrane receptor	Activated	2.236	2.15E-08	IFIT1, IFIT3, ISG15, MX1, STAT1
IFNB1	cytokine	Activated	2.623	1.35E-13	IFIT1, IFIT2, IFIT3, ISG15, MX1, PML, STAT1
IFNG	cytokine	Activated	3.096	4.58E-17	EIF2AK2, GBP1, HLA-A, HLA-B, ICAM1, IFIT1, IFIT3, INS, ISG15, MX1, PML, STAT1, TAP1, TYMP
IFNA2	cytokine	Activated	3.148	1.26E-16	EIF2AK2, GBP1, IFIT1, IFIT2, IFIT3, ISG15, MX1, PML, STAT1, UBE2L6
EIF2AK2*	kinase	Activated	2.412	5.44E-10	EIF2AK2, IFIT1, ISG15, SAMHD1, STAT1, UBE2L6
EBI3	cytokine	Activated	2.449	9.50E-13	B2M, HLA-A, HLA-B, ICAM1, STAT1, TAP1
IL1RN	cytokine	Inhibited	-2.433	2.16E-09	GBP1, ICAM1, IFIT3, INS, MX1, PML

*Expression fold change 1.927.

## Supplementary Materials

The following are available online at https://www.mdpi.com/2076-2607/8/2/295/s1: Table S1: Proteins with >1.5-fold (log2) difference in CVB4-infected compared to uninfected SC-β cells at 72 h. Figure S1: Assessment of perifusion and total insulin in CVB4-infected SC-β cells in an independent experiment from that shown in Figure 5. Table S2: Supplementary data file transcriptome.xlsx
