# Supplementary material for: Proteomic and Transcriptional Profiles of Human Stem Cell-Derived β Cells Following Enteroviral Challenge
Source: Microorganisms. 2020 Feb 20;8(2):295. doi: 10.3390/microorganisms8020295 (PMC7074978; doi:10.3390/microorganisms8020295)
Supplement: Supplementary file 1 [file microorganisms-08-00295-s001.zip › Supplementary Files_703371/Table S1 and Figure S1_rev 021120.docx]

**Table S1:** Proteins with >1.5-fold (log2) difference in CVB4-infected compared to uninfected SC-β cells at 72 h.

| **Table S1. Proteins with >1.5-fold difference in CVB4-infected compared to uninfected SC-β cells at 72 h.** | | | |
| --- | --- | --- | --- |
| **= -LOG (P-value)** | **Fold Difference (log2 transformed)** | **Protein IDs** | **Gene name/Protein name** |
| 3.716613 | 9.274271 | Q12912 | *LRMP*/Lymphoid-restricted membrane protein |
| 4.631188 | 7.654553 | P20591 | *MX1*/Interferon-induced GTP-binding protein Mx1 |
| 4.240699 | 7.094229 | P08292 | *POLG_CXB4J*/Genome polyprotein |
| 6.482044 | 4.857399 | P10145 | *CXCL8*/Interleukin-8 |
| 3.920683 | 4.272551 | Q04941 | *PLP2*/Proteolipid protein 2 |
| 2.475191 | 4.102964 | P07996 | *THBS1*/Thrombospondin-1 |
| 1.337612 | 4.056866 | Q4VCS5 | *AMOT*/Angiomotin |
| 6.140962 | 4.048493 | P30480 | *HLA-B*/HLA class I histocompatibility antigen |
| 5.256661 | 3.949762 | P19525 | *EIF2AK2*/Interferon-induced, double-stranded RNA-activated protein kinase |
| 4.892307 | 3.847692 | O14879 | *IFIT3*/Interferon-induced protein with tetratricopeptide repeats 3 |
| 3.399121 | 3.729094 | P09914 | *IFIT1*/Interferon-induced protein with tetratricopeptide repeats 1 |
| 1.460734 | 3.116309 | P10412 | *HIST1H1E*/Histone H1.4;Histone H1.1 |
| 2.110538 | 2.956483 | Q9P2X0 | *DPM3*/Dolichol-phosphate mannosyltransferase subunit 3 |
| 1.573859 | 2.937955 | Q9UGI8 | *TES*/Testin |
| 2.619759 | 2.925711 | O14735 | *CDIPT*/CDP-diacylglycerol--inositol 3-phosphatidyltransferase |
| 1.369625 | 2.843 | Q15120 | *PDK3*/[Pyruvate dehydrogenase (acetyl-transferring)] kinase isozyme 3 |
| 1.26255 | 2.835231 | Q9NZ08 | *ERAP1*/Endoplasmic reticulum aminopeptidase 1 |
| 1.437613 | 2.820944 | O75431 | *MTX2*/Metaxin-2 |
| 1.557091 | 2.820487 | Q13445 | *TMED1*/Transmembrane emp24 domain-containing protein 1 |
| 1.753157 | 2.791638 | O95786 | *DDX58*/Probable ATP-dependent RNA helicase DDX58 |
| 1.239624 | 2.688893 | Q8NHP8 | *PLBD2*/Putative phospholipase B-like 2 |
| 1.054095 | 2.645543 | Q9Y512 | *SAMM50*/Sorting and assembly machinery component 50 homolog |
| 1.720049 | 2.598983 | Q92506 | *HSD17B8*/Estradiol 17-beta-dehydrogenase 8 |
| 1.051674 | 2.551734 | O95470 | *SGPL1*/Sphingosine-1-phosphate lyase 1 |
| 1.717618 | 2.536067 | O14828 | *SCAMP3*/Secretory carrier-associated membrane protein 3 |
| 3.624925 | 2.533079 | O15042 | *U2SURP*/U2 snRNP-associated SURP motif-containing protein |
| 1.04885 | 2.518101 | Q96I99 | *SUCLG2*/Succinyl-CoA ligase [GDP-forming] subunit beta, mitochondrial |
| 4.779319 | 2.515262 | O00116 | *AGPS*/Alkyldihydroxyacetonephosphate synthase, peroxisomal |
| 1.143659 | 2.43997 | Q8TBC4 | *UBA3*/NEDD8-activating enzyme E1 catalytic subunit |
| 2.700888 | 2.416928 | Q14696 | *MESDC2*/LDLR chaperone MESD |
| 5.500638 | 2.377149 | P42224 | *STAT1*/Signal transducer and activator of transcription 1-alpha/beta |
| 1.852953 | 2.36031 | O00743 | *PPP6C*/Serine/threonine-protein phosphatase 6 catalytic subunit; |
| 1.738587 | 2.349311 | P82673 | *MRPS35*/28S ribosomal protein S35, mitochondrial |
| 2.234435 | 2.341794 | Q9NS69 | *TOMM22*/Mitochondrial import receptor subunit TOM22 homolog |
| 5.056107 | 2.310655 | P30453 | *HLA-A*/HLA class I histocompatibility antigen, A-34 alpha chain |
| 1.609513 | 2.2201 | P50583 | *NUDT2*/Bis(5-nucleosyl)-tetraphosphatase [asymmetrical] |
| 1.084362 | 2.161926 | O76031 | *CLPX*/ATP-dependent Clp protease ATP-binding subunit clpX-like, |
| 1.489283 | 2.152734 | P30038 | *ALDH4A1*/Delta-1-pyrroline-5-carboxylate dehydrogenase, mitochondrial |
| 1.217907 | 2.135079 | Q8TD08 | *MAPK15*/Mitogen-activated protein kinase 15 |
| 1.240598 | 2.082697 | Q86TI2 | *DPP9*/Dipeptidyl peptidase 9 |
| 2.016421 | 2.06303 | P25685 | *DNAJB1*/DnaJ homolog subfamily B member 1 |
| 1.748206 | 2.059379 | Q8WW59 | *SPRYD4*/SPRY domain-containing protein 4 |
| 1.397296 | 2.02298 | P09341 | *CXCL1*/Growth-regulated alpha protein |
| 1.969093 | 1.992005 | O95486 | *SEC24A*/Protein transport protein Sec24A |
| 1.138286 | 1.990897 | P32322 | *PYCR1*/Pyrroline-5-carboxylate reductase 1, mitochondrial |
| 1.447696 | 1.987773 | Q96M27 | *PRRC1*/Protein PRRC1 |
| 1.148267 | 1.986922 | P06730 | *EIF4E*/Eukaryotic translation initiation factor 4E |
| 1.304541 | 1.982758 | Q5VWZ2 | *LYPLAL1*/Lysophospholipase-like protein 1 |
| 4.530025 | 1.978498 | Q9H3Z4 | *DNAJC5*/DnaJ homolog subfamily C member 5 |
| 2.042418 | 1.966346 | P80162 | *CXCL6*/C-X-C motif chemokine 6 |
| 1.129672 | 1.930754 | O43395 | *PRPF3*/U4/U6 small nuclear ribonucleoprotein Prp3 |
| 1.141472 | 1.904081 | Q567U6 | *CCDC93*/Coiled-coil domain-containing protein 93 |
| 1.132874 | 1.865278 | Q86UE4 | *MTDH*/Protein LYRIC |
| 1.675613 | 1.859769 | Q9H0D6 | *XRN2*/5-3 exoribonuclease 2 |
| 2.83522 | 1.817125 | P41732 | *TSPAN7*/Tetraspanin-7 |
| 2.04719 | 1.777068 | Q99442 | *SEC62*/Translocation protein SEC62 |
| 1.264019 | 1.767001 | Q9UPT5 | *EXOC7*/Exocyst complex component 7 |
| 1.218679 | 1.766781 | O75436 | *VPS26A*/Vacuolar protein sorting-associated protein 26A |
| 1.713295 | 1.764956 | Q8IYS1 | *PM20D2*/Peptidase M20 domain-containing protein 2 |
| 1.142742 | 1.74316 | Q9NQT8 | *KIF13B*/Kinesin-like protein KIF13B |
| 1.122945 | 1.737365 | Q9H254 | *SPTBN4*/Spectrin beta chain, non-erythrocytic 4 |
| 1.725622 | 1.712251 | P48728 | *AMT*/Aminomethyltransferase, mitochondrial |
| 1.552387 | 1.708257 | Q14160 | *SCRIB*/Protein scribble homolog |
| 2.222998 | 1.679876 | Q8NAF0 | *ZNF579*/Zinc finger protein 579 |
| 1.132415 | 1.67574 | P30626 | *SRI*/Sorcin |
| 2.666506 | 1.639322 | Q96AX1 | *VPS33A*/Vacuolar protein sorting-associated protein 33A |
| 1.46788 | 1.620991 | P62072 | *TIMM10*/Mitochondrial import inner membrane translocase subunit Tim10 |
| 1.623454 | 1.618961 | P12830 | *CDH1*/Cadherin-1 |
| 1.253111 | 1.608814 | O43865 | *AHCYL1*/Putative adenosylhomocysteinase 2 |
| 1.183372 | 1.605277 | Q8IXB1 | *DNAJC10*/DnaJ homolog subfamily C member 10 |
| 1.551521 | 1.585485 | P82933 | *MRPS9*/28S ribosomal protein S9, mitochondrial |
| 1.638593 | 1.582642 | Q13586 | *STIM1*/Stromal interaction molecule 1 |
| 5.553053 | 1.581921 | Q8N2U0 | *TMEM256*/Transmembrane protein 256 |
| 1.269093 | 1.553296 | Q8N4H5 | *TOMM5*/Mitochondrial import receptor subunit TOM5 homolog |
| 1.972669 | 1.535308 | P12882 | *MYH1*/Myosin-1 |
| 1.418282 | 1.523942 | Q9NRY4 | *ARHGAP35*/Rho GTPase-activating protein 35 |
| 1.522091 | 1.521898 | Q8NBQ5 | *HSD17B11*/Estradiol 17-beta-dehydrogenase 11 |
| 3.517185 | -1.50455 | P56211 | *ARPP19*/cAMP-regulated phosphoprotein 19 |
| 4.784992 | -1.51564 | P26599 | *PTBP1*/Polypyrimidine tract-binding protein 1 |
| 3.281665 | -1.51815 | P20962 | *PTMS*/Parathymosin |
| 2.467302 | -1.53311 | P52569 | *SLC7A2*/Cationic amino acid transporter 2 |
| 4.304682 | -1.56862 | P16949 | *STMN1*/Stathmin |
| 2.389681 | -1.57184 | Q8IYB3 | *SRRM1*/Serine/arginine repetitive matrix protein 1 |
| 4.046858 | -1.57327 | O43670 | *ZNF207*/BUB3-interacting and GLEBS motif-containing protein ZNF207 |
| 3.559595 | -1.58462 | Q96PK6 | *RBM14*/RNA-binding protein 14 |
| 1.744927 | -1.62343 | Q9UI15 | *TAGLN3*/Transgelin-3 |
| 1.155406 | -1.62418 | O14936 | *CASK*/Peripheral plasma membrane protein CASK |
| 3.669369 | -1.62733 | Q15843 | *NEDD8*/NEDD8 |
| 6.290551 | -1.63503 | P27816 | *MAP4*/Microtubule-associated protein 4 |
| 2.505924 | -1.65015 | Q969G3 | *SMARCE1*/SWI/SNF-related matrix-associated actin-dependent regulator of chromatin subfamily E member 1 |
| 4.795634 | -1.65514 | P98179 | *RBM3*/RNA-binding protein 3 |
| 6.809146 | -1.66226 | P14209 | *CD99*/CD99 antigen |
| 2.597041 | -1.66236 | P05204 | *HMGN2*/Non-histone chromosomal protein HMG-17 |
| 1.688854 | -1.66734 | Q9NTZ6 | *RBM12*/RNA-binding protein 12 |
| 1.561533 | -1.67305 | P53990 | *IST1*/IST1 homolog |
| 4.005021 | -1.70271 | Q9Y2S6 | *TMA7*/Translation machinery-associated protein 7 |
| 1.544645 | -1.72267 | O43504 | *LAMTOR5*/Regulator complex protein LAMTOR5 |
| 1.527765 | -1.72385 | Q8WZA9 | *IRGQ*/Immunity-related GTPase family Q protein |
| 2.093691 | -1.72888 | P12081 | *HARS*/Histidine--tRNA ligase, cytoplasmic |
| 2.755207 | -1.74202 | Q2TAA2 | *IAH1*/Isoamyl acetate-hydrolyzing esterase 1 homolog |
| 1.090424 | -1.74883 | Q96K17 | *BTF3L4*/Transcription factor BTF3 homolog 4 |
| 2.737724 | -1.76398 | Q5BKZ1 | *ZNF326*/DBIRD complex subunit ZNF326 |
| 3.469616 | -1.78045 | P35241 | *RDX*/Radixin |
| 1.552344 | -1.78718 | Q99519 | *NEU1*/Sialidase-1 |
| 5.862311 | -1.80128 | P67809 | *YBX1*/Nuclease-sensitive element-binding protein 1 |
| 3.604416 | -1.80854 | Q09666 | *AHNAK*/Neuroblast differentiation-associated protein AHNAK |
| 4.541386 | -1.82579 | Q6PKG0 | *LARP1*/La-related protein 1 |
| 1.167075 | -1.84193 | O76024 | *WFS1*/Wolframin |
| 4.168806 | -1.84395 | P28370 | *SMARCA1*/Probable global transcription activator SNF2L1 |
| 2.602539 | -1.86254 | Q7L2H7 | *EIF3M*/Eukaryotic translation initiation factor 3 subunit M |
| 1.916615 | -1.86631 | P12270 | *TPR*/Nucleoprotein TPR |
| 3.1255 | -1.88163 | Q14739 | *LBR*/Lamin-B receptor |
| 4.348886 | -1.90015 | P50897 | *PPT1*/Palmitoyl-protein thioesterase 1 |
| 3.248227 | -1.91621 | P18085 | *ARF4*/ADP-ribosylation factor 4 |
| 1.464788 | -1.93958 | O43399 | *TPD52L2*/Tumor protein D54 |
| 2.474809 | -1.95561 | P51532 | *SMARCA4*/Transcription activator BRG1 |
| 1.841048 | -1.97298 | O43583 | *DENR*/Density-regulated protein |
| 2.812767 | -1.97839 | Q8WW12 | *PCNP*/PEST proteolytic signal-containing nuclear protein |
| 2.57751 | -2.02588 | O43768 | *ENSA*/Alpha-endosulfine |
| 2.306232 | -2.03975 | Q9UMX0 | *UBQLN1*/Ubiquilin-1 |
| 2.160607 | -2.0448 | Q9UQ35 | *SRRM2*/Serine/arginine repetitive matrix protein 2 |
| 3.825954 | -2.04747 | P49720 | *PSMB3*/Proteasome subunit beta type-3 |
| 1.58204 | -2.05973 | P61513 | *RPL37A*/60S ribosomal protein L37a |
| 2.015909 | -2.13095 | Q13442 | *PDAP1*/28 kDa heat- and acid-stable phosphoprotein |
| 1.747385 | -2.18339 | Q14157 | *UBAP2L*/Ubiquitin-associated protein 2-like |
| 1.560967 | -2.19525 | Q9UPN3 | *MACF1*/Microtubule-actin cross-linking factor 1, isoforms 1/2/3/5 |
| 2.011486 | -2.19859 | Q16623 | *STX1A*/Syntaxin-1A |
| 1.565166 | -2.21867 | Q02952 | *AKAP12*/A-kinase anchor protein 12 |
| 3.098782 | -2.22498 | P62318 | *SNRPD3*/Small nuclear ribonucleoprotein Sm D3 |
| 1.233665 | -2.24326 | O15212 | *PFDN6*/Prefoldin subunit 6 |
| 2.745286 | -2.27503 | Q16576 | *RBBP7*/Histone-binding protein RBBP7 |
| 1.195428 | -2.32254 | P63220 | *RPS21*/40S ribosomal protein S21 |
| 6.38836 | -2.34426 | P22676 | *CALB2*/Calretinin |
| 2.142486 | -2.35728 | Q9UHB6 | *LIMA1*/LIM domain and actin-binding protein 1 |
| 1.065555 | -2.36188 | O95777 | *LSM8*/U6 snRNA-associated Sm-like protein LSm8 |
| 5.049106 | -2.49807 | Q14683 | *SMC1A*/Structural maintenance of chromosomes protein 1A |
| 1.780003 | -2.53672 | Q53FA7 | *TP53I3*/Quinone oxidoreductase PIG3 |
| 2.636718 | -2.55098 | Q92804 | *TAF15*/TATA-binding protein-associated factor 2N |
| 4.717435 | -2.65843 | P15104 | *GLUL*/Glutamine synthetase |
| 2.478724 | -2.92936 | P48637 | *GSS*/Glutathione synthetase |
| 2.634573 | -2.95873 | P17677 | *GAP43*/Neuromodulin |
| 5.701657 | -2.97861 | P49903 | *SEPHS1*/Selenide, water dikinase 1 |
| 1.849698 | -3.04046 | Q9H9Q2 | *COPS7B*/COP9 signalosome complex subunit 7b |
| 3.312902 | -3.2471 | O15066 | *KIF3B*/Kinesin-like protein KIF3B |
| 2.919343 | -3.96133 | Q92734 | *TFG*/Protein TFG |
| 2.514759 | -4.05724 | P47914 | 60S ribosomal protein L29 |


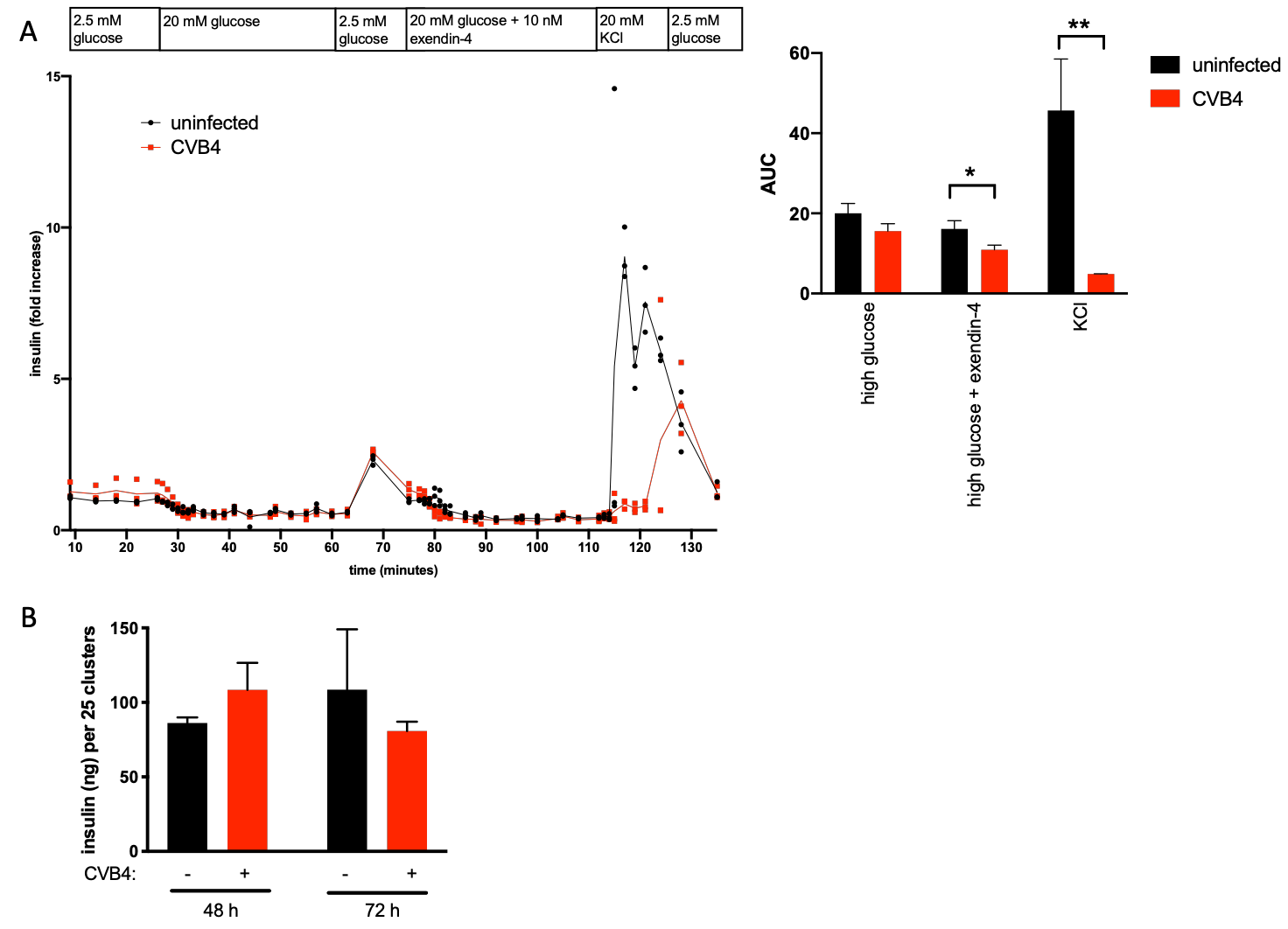


**Figure S1. Assessment of perifusion and total insulin in CVB4-infected SC-β cells in an independent experiment from that shown in Figure 5.** **A.** Cells were examined by perifusion analysis at 48 h post-infection to measure insulin secretion. The data are normalized to basal insulin secretion values measured prior to challenge with high glucose. Insulin secretion in response to KCl (20 mM) is diminished in CVB4-infected cells compared to uninfected cells (left panel). Insulin secretion in response to high glucose (20 mM) and high glucose plus exendin-4 (10 nM) is not observed in this experiment. Perifusion data are also presented as the area under the curve (AUC) for insulin secretion under conditions of high glucose (27-60 min), high glucose + exendin-4 (77-108 min), and KCl (112-121 min) (right panel). The mean +/- S.D. for triplicate samples is shown. *, *P*<0.05; **, *P*<0.01, Student’s t-test. **B.** Total insulin content does not differ between uninfected and CVB4-infected cells at either 48 or 72 h. Error bars indicate the S.D. of values from triplicate wells measured in duplicate. Differences between uninfected and infected cells are not statistically significant.
